# In-Depth Analysis of Unmodulated Visible Light Positioning Using the Iterated Extended Kalman Filter

**DOI:** 10.3390/s19235198

**Published:** 2019-11-27

**Authors:** Robin Amsters, Eric Demeester, Nobby Stevens, Peter Slaets

**Affiliations:** 1 KU Leuven, Department of Mechanical Engineering, 3000 Leuven, Belgium; eric.demeester@kuleuven.be (E.D.); peter.slaets@kuleuven.be (P.S.); 2 KU Leuven, Department of Electrical Engineering, 3000 Leuven, Belgium; nobby.stevens@kuleuven.be

**Keywords:** Kalman Filter, Visible Light Positioning, Sensor Fusion, Indoor Positioning

## Abstract

Indoor positioning with visible light has become increasingly important in recent years. Usually, light sources are modulated at high speeds in order to wirelessly transmit data from the fixtures to a receiver. The accuracy of such systems can range from a few decimeters to a few centimeters. However, additional modulation hardware is required for every light source, thereby increasing cost and system complexity. This paper investigates the use of unmodulated light for indoor positioning. Contrary to previous work, a Kalman filter is used instead of a particle filter to decrease the computational load. As a result, the update rate of position estimation can be higher. Additionally, more resources could be made available for other tasks (e.g., path planning for autonomous robots). We evaluated the performance of our proposed approach through simulations and experiments. The accuracy depends on a number of parameters, but is generally lower than 0.5 m. Moreover, temporary occlusion of the receiver can be compensated in most cases.

## 1. Introduction

Indoor positioning is not a new problem. Ever since the widespread adoption of the global positioning system (GPS), researchers and industry professionals have searched for a similar, ubiquitous positioning solution for indoor environments. However, due to the large disparity in layouts for indoor spaces, there likely will never be a single “best” indoor positioning system (IPS). Every solution has its own advantages and disadvantages [1]. Multiple taxonomies have been proposed for the field of indoor positioning [2]. One approach is to categorize IPS into infrastructure-based and infrastructure-free systems [3]. As the name suggests, infrastructure-based systems require the deployment of dedicated positioning hardware (such as ultrawideband [4] or Bluetooth transmitters [5]). On the other hand, infrastructure-free systems leverage existing position-dependent signals (such as Wi-Fi signal strength [6]) to estimate the user’s location. Such systems are therefore significantly less expensive than those that require dedicated hardware. However, it is more challenging to achieve high accuracy and robustness compared to infrastructure-based systems.

LED illumination is becoming more and more widespread, both outdoors and indoors. LED fixtures are energy-efficient and have long lifetimes and low maintenance costs [7]. The LED lighting market is therefore expected to surpass 100 billion USD in 2024 [8]. Besides cost reduction, LED fixtures present other opportunities as well, most notably in wireless communications. It is possible to modulate the intensity of LED fixtures at speeds imperceptible to the human eye. These modulations can be detected by a photodiode or camera, thus establishing a wireless communication link between the fixtures and the receiver [9]. This principle is also known as visible light communication (VLC), and has a number of advantages over traditional radio frequency (RF) based communication. For example, the potential bandwidth is significantly larger than RF communication. Additionally, there is no electromagnetic interference and the required transmitting/receiving hardware is relatively low-cost [10]. Visible light can also be used as a means of position estimation, which is sometimes referred to as Visible Light Positioning (VLP) [11]. Light sources already installed in buildings then have a dual purpose of both illumination and positioning. A completely dedicated infrastructure is therefore not required, as the light fixtures and power supplies are often already present. One could even envision a system that can be used for both communication and positioning simultaneously. In such a system, however, resource allocation between the positioning and communications portions is an additional challenge [12].

Regardless of whether the system performs only communication, only positioning or both, some additional hardware is required to modulate the light intensity at a high frequency. A smaller subfield of VLP exists that attempts to use light as a signal of opportunity. In this case, no extra hardware is added to the fixtures, and the light intensity is continuous and constant. We call this approach “Unmodulated Visible Light Positioning” (UVLP). Light is an inherently position-dependent signal: when one moves closer to a light source, a higher intensity value is perceived and vice versa. The measured light intensity can therefore be used to infer the proximity of the receiver to one or more light sources. In this work, we investigate the use of an iterated extended Kalman filter (IEKF) for positioning of a mobile robot with unmodulated light. Encoder measurements are used in the prediction step, and the estimate is later updated by light measurements from one of more photodiodes. We evaluated our proposed approach in both a simulation and an experimental environment.

The rest of the paper is structured as follows. Section 2 presents related work and the main contributions of this paper. Section 3 elaborates on the materials and methods used in this work. Simulation and experimental results are presented in Section 4 and Section 5, respectively. These results are discussed in more detail and compared to literature in Section 6. Finally, a conclusion is drawn in Section 7.

## 2. Related Work

In conventional Visible Light Positioning, light intensity is generally captured by either a photodiode (PD) or a camera. When using a PD, one can use the photocurrent to determine the distance to the transmitters via a signal strength model. This method is relatively straightforward, yet sensitive to fluctuations in light intensity, which can occur for example with dimming [11]. Alternatively, one can use time of flight or round trip time to obtain the transmitter–receiver distance. Distance measurements based on signal travel time are robust to changes in signal strength, yet require very accurate time synchronization [11]. Additionally, high-speed sampling is often required. However, more recently, methods have been proposed to relax some of these requirements [13]. Image sensors can be seen as a collection of photodiodes, and therefore provide more information. Consequently, robustness and high accuracy are easier to achieve [7]. On the other hand, because images are a lot denser in information, processing is more challenging and typically induces higher latencies.

UVLP can exploit either PD or camera measurements as well, similar to conventional VLP. One of the first examples of UVLP can be found in the work of Facchinetti, Tièche, and Hügli [14]. Ceiling structures such as fluorescent light fixtures are used as landmarks for computer-vision based localization. Light fixtures are relatively easy to detect, as they typically have high intensity values in an image. Therefore, computationally expensive algorithms that are typically associated with computer vision can be simplified [15]. Additionally, features on the ceiling are not as easily obstructed as other natural landmarks. As a result of these advantages, several publications on the use of light fixtures as landmarks for localization or SLAM (simultaneous localization and mapping) are available in the literature [16,17,18]. Compared to photodiode-based approaches, camera-based systems still require more computational power and typically induce higher latencies. Additionally, image sensors are significantly more expensive than photodiodes.

When using a photodiode for VLP, the light intensity of each source is typically modulated in a way that is unique for each light source. It should be possible to demultiplex the combined signal at the receiver, for example, via code division or frequency division [19]. UVLP cannot separate the signal into the components emitted by the individual light sources. A single intensity value is therefore not often used for positioning. A set of intensity measurements can be utilized by classification algorithms, as is the case in the work of Ravi and Iftode [20]. More often, light intensity is combined with other sensor data to improve classification [21,22].

One could also rely on the inherent manufacturing differences between light sources, in order to extract unique features for every transmitter. The Littel system by Zhang and Zhang uses unmodified fluorescent lights to determine the location of an Android smartphone [23]. Due to manufacturing tolerances, the driver of a fluorescent light introduces a light-dependent resonance frequency, which is typically in the 80–160 kHz range. To sample such a high frequency signal with a commercial smartphone, the rolling shutter principle from camera-based VLP is not sufficient. The authors noted that the analog bandwidth of the camera is much larger than the digital sampling rate and proposed a way to recover even higher frequency signals through the known aliasing properties. This process does require very accurate calibration of the camera’s sampling frequency. Contrary to Littel, the LiLoc system by Wang et al. [24] determines visual features to identify unmodified light sources, rather than frequency features. Images from the front-facing camera of a smartphone are also processed, yet in this case unique radiance patterns are detected in the light fixture projection. Access to the RAW pixel data is required, which is not available on every smartphone. Additionally, the same features may appear differently on different devices.

Finally, Bayesian estimators have also been used for UVLP. Generally, pedestrian dead-reckoning (PDR) is applied to obtain the displacement of the mobile unit, which is fused by a particle filter with an observation model based on light peak detection [25,26,27]. However, particle filters can be computationally expensive. In our previous work, we proposed the use of an iterated extended Kalman filter for UVLP [28]. The reduced computational load makes it possible to be embedded in smaller devices. The approach was evaluated exclusively in a simulation environment. In this paper, we extend that work and provide the following contributions:

- We provide much more in-depth simulations, to better characterize the limits of this approach. Among other factors, we investigate the influence of:
  - Partial shadowing
  - Random trajectories
  - Robot movement speed
  - Imperfect calibration
- We validated the approach with experimental data.

## 3. Materials and Methods

### 3.1. IEKF Formulation

**Figure 1:**
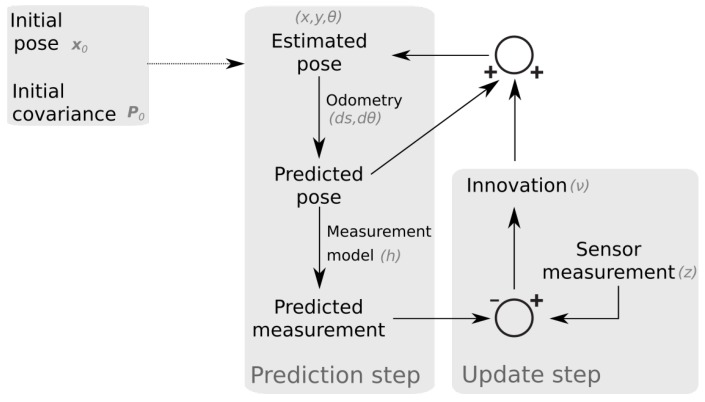
Schematic overview of the proposed positioning approach.

This work uses an iterated extended Kalman filter to estimate the pose (position and orientation) of a mobile robot. Figure 1 provides a graphical overview of the algorithm. A detailed explanation is elaborated in this section.

The standard formulation of the Kalman filter estimates the state of a dynamical system, if the state transition and the measurement probabilities are linear. The extended Kalman filter (EKF) is able to perform state estimation for non-linear models, by linearizing around the current estimate. The IEKF can improve performance in the case of a highly non-linear measurement model by making local iterations of the update step [29]. The local iterations are stopped when subsequent estimates are close enough in state space, or when a maximum number of iterations is reached.

The Kalman filter and its derivatives all work in two steps. The first step or “prediction” step makes a prediction of the next state based on the previous state and a state transition model (also called the “motion model”):

(1) $$\begin{array}{ccc} {\hat{x}}_{n|n-1} & = & f\left({\tilde{x}}_{n-1|n-1},u_{n}\right)\\ {\hat{P}}_{n|n-1} & = & F_{n}{\tilde{P}}_{n-1|n-1}F_{n}^{T}+Q_{n} \end{array}$$

where x is the state vector, u is the control input vector (odometry measurements in this work), P is the state covariance matrix, F is the state transition matrix, and Q is the process noise covariance matrix. F is obtained by taking the Jacobian matrix of the state transition model with respect to the state vector $\left(F_{n}=\frac{\partial f(x,u)}{\partial x}{|}_{{\hat{x}}_{n-1|n-1},u_{n}}\right)$. In this paper, boldface symbols are used to represent matrix quantities (e.g., F). A tilde symbol is used for estimated quantities (e.g., ${\tilde{x}}_{n}$), and a hat symbol is used for predicted quantities (e.g., ${\hat{y}}_{n}$). Subscripts *n*, and n−1 are used to denote time steps. In the case of a differential drive mobile robot, the motion model can be defined as [30]:

(2) $$\begin{array}{cc} {\hat{x}}_{n|n-1} & =\left\{\begin{array}{c} {\hat{x}}_{n|n-1}={\tilde{x}}_{n-1|n-1}+\Delta s_{n}\cos\left({\tilde{\theta }}_{n-1|n-1}+\Delta \theta_{n}\right)\\ {\hat{y}}_{n|n-1}={\tilde{y}}_{n-1|n-1}+\Delta s_{n}\sin\left({\tilde{\theta }}_{n-1|n-1}+\Delta \theta_{n}\right)\\ {\hat{\theta }}_{n|n-1}={\tilde{\theta }}_{n-1|n-1}+\Delta \theta_{n} \end{array}\right. \end{array}$$

where $\Delta s_{n}$ is the translation relative to the previous position and $\Delta \theta_{n}$ is the rotation relative to the previous position.

In principle, one could continue to use the prediction step indefinitely. However, each prediction has a slight error; therefore, the total estimation error grows without bounds and the uncertainty can only increase (all terms in the covariance equation of Equation (1) are positive definitive). The Kalman filter therefore also employs a second step, which is known as the “update” step. Based on the predicted state, a measurement is predicted using a so-called “measurement model”. This predicted measurement is then compared to sensor data. The difference between the real and the predicted measurement is new information (also known as the “innovation”), which is weighed with the predicted state in order to obtain a new estimate. The weighing factor is called the “Kalman gain” and is calculated based on the relative uncertainties of the motion and measurement model. Mathematically, the update step is formulated as follows:

(3) $$\begin{array}{ccc} \nu_{n} & = & y_{n}-h\left({\hat{x}}_{n|n-1}\right)\\ K_{n} & = & {\hat{P}}_{n|n-1}H_{n}^{T}S_{n}^{-1}\\ S_{n} & = & H_{n}{\hat{P}}_{n|n-1}H_{n}^{T}+R_{n}\\ {\tilde{x}}_{n|n} & = & {\hat{x}}_{n|n-1}+K_{n}\nu_{n}\\ {\tilde{P}}_{n|n} & = & \left(I-K_{n}H_{n}\right){\hat{P}}_{n|n-1} \end{array}$$

where ν is the innovation, y is the measurement, $h({\hat{x}}_{n|n-1})$ is the predicted measurement (which in this work is the sampled voltage of the receiver), K is the Kalman gain, H is the state observation matrix, R is the measurement noise covariance matrix, and I is the identity matrix. H is obtained by taking the Jacobian matrix of the measurement model with respect to the state vector $\left(H_{n}=\frac{\partial h(x)}{\partial x}{|}_{{\hat{x}}_{n|n-1}}\right)$.

**Figure 2:**
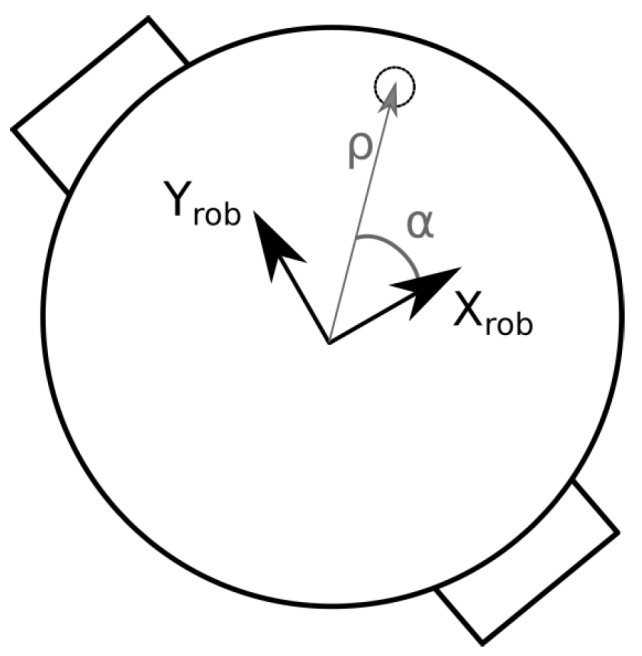
Coordinate frame conventions for receiver positions.

The measurement model is based on a Lambertian radiation model for the transmitter and a photodiode with a trans-impedance amplifier (TIA) as receiver [31]. Only the line-of-sight components are considered in the measurement model. In the case a single receiver is used, the receiver coordinates should coincide with the robot origin, thus removing the need for coordinate frame transformations. In the case multiple receivers are used, it is desirable to express the receiver position relative to the robot frame in polar coordinates. With this formulation, the robot orientation θ becomes a variable in the measurement model, and it can be estimated by the Kalman filter (instead of only being able to estimate *x*- and *y*-coordinates with the previous formulation). The receiver position is thus expressed as [28]:

(4) $$P_{Rx,j}=\left[\begin{array}{c} x_{Rx,j}\\ y_{Rx,j}\\ z_{Rx,j} \end{array}\right]=\left[\begin{array}{c} x_{rob}+\rho_{j}\cos\left(\alpha_{j}+\theta_{rob}\right)\\ y_{rob}+\rho_{j}\sin\left(\alpha_{j}+\theta_{rob}\right)\\ z_{rob} \end{array}\right]$$

where $P_{Rx,j}$ is the position of receiver *j*, $\alpha_{j}$ is the polar angle of receiver *j*, and $\rho_{j}$ is the radial coordinate of receiver *j* (see Figure 2). The subscript rob is used to denote the coordinates of the robot.

**Figure 3:**
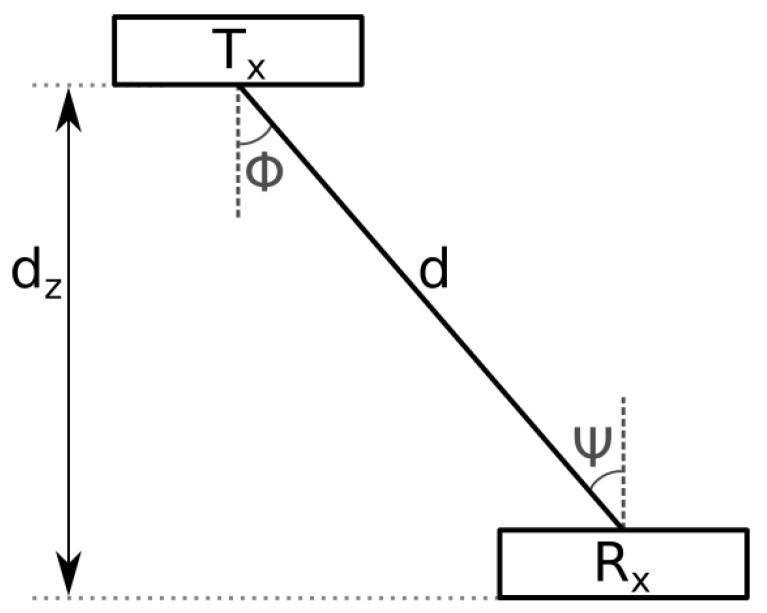
Overview of a VLC link.

The voltage sampled at the output of the receiver is used as the measurement for the Kalman filter ($y_{n}$ in Equation (3)) and is given by [28,31]:

(5) $$V_{R}=\left\{\begin{array}{cc} \left[\sum_{i=1}^{n_{T_{x}}}C_{i}\frac{\cos^{m}\left(\phi_{i}\right)\cos\left(\psi_{i}\right)}{d_{i}^{2}}\right]+G\left(\left[\sum_{i=1}^{n_{T_{x}}}N_{S,i}\right]+N_{bg}+N_{T}\right) & 0\leq \psi \leq \psi_{c}\\ G\left(N_{bg}+N_{T}\right) & \psi >\psi_{c} \end{array}\right.$$

where $V_{R}$ is the output voltage of the receiver; $n_{T_{x}}$ is the number of transmitters, *m* is the Lambertian emission order of the LED; ϕ and ψ are the irradiance and incidence angles with respect to the light source, respectively (see Figure 3); *d* is the Euclidean distance between transmitter and receiver; *G* is the transimpedance of the receiver; $N_{S,i}$ is shot noise current on the received power; $N_{bg}$ is shot noise current due to background illumination; $N_{T}$ is thermal noise current [31]; and $\psi_{c}$ is the field of view of the photodetector. Finally, $C_{i}$ in Equation (5) is a constant that contains all non-position dependent parameters [28]:

(6) $$C_{i}=\frac{\left(m_{i}+1\right)GAR_{P}}{2\pi }\int_{\lambda_{min}}^{\lambda_{max}}P_{T,i}\left(\lambda \right)S_{R}^{\prime }\left(\lambda \right)d\lambda \;,$$

where *A* is the active area of the photodiode; $R_{P}$ is the peak responsivity of the photodiode; $\lambda_{min}$ and $\lambda_{max}$ are the minimum and maximum wavelengths of light for which the photodiode produces a current, respectively; $P_{T,i}\left(\lambda \right)$ is the transmitted optical power of transmitter *i*; and $S_{R}^{\prime }\left(\lambda \right)$ is the normalized spectral response of the photodiode.

### 3.2. Simulation Environment

**Figure 4:**
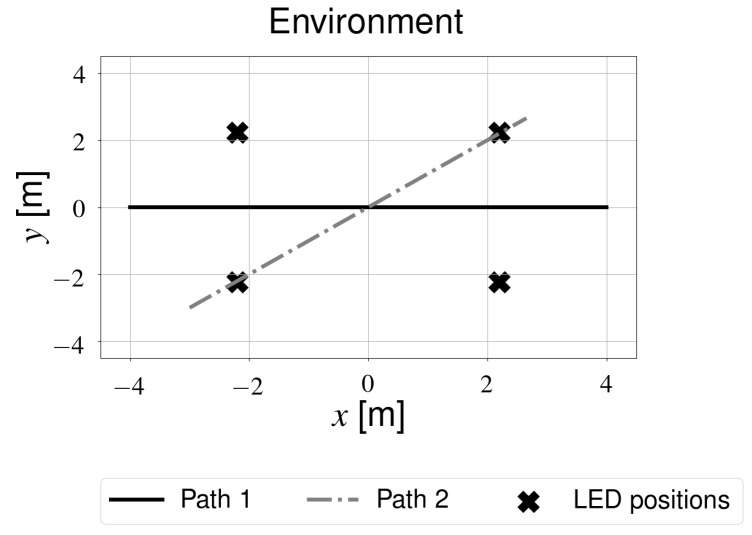
Paths that were simulated or approximately driven during experiments.

The simulation environment was modeled after the experimental setup (see Section 3.3). The datasheets of the various components (see Table 1) were consulted to determine parameters related to the receiver and transmitters. Additionally, several assumptions were made for the more general parameters (Table 2). The light sources were placed in a square arrangement, as was approximately the case during experiments. During most experiments and simulations, one of two pre-defined trajectories was followed (see Figure 4), unless specified otherwise.

### 3.3. Experimental Setup

**Figure 5:**
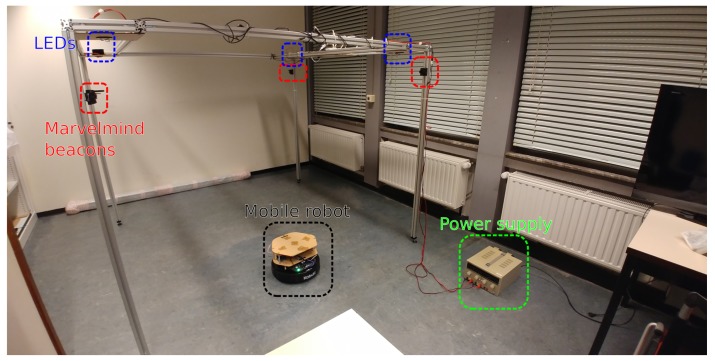
Experimental setup.

To verify the proposed approach, experiments were performed in a lab environment (see Figure 5). The robot was equipped with a custom sensor platform, and data of the various sensors were collected. Position estimation was performed in post-processing. Therefore, the experimental results are not real-time positioning results. However, we would like to point out that the proposed algorithm is not very costly from a computational point of view (see Section 5). The choice to perform the estimation in post-processing was mostly motivated by the fact that this speeds up development significantly, as data can be recorded once and then used throughout the development of the filter. To ensure proper separation between calibration data and validation data, some datasets were only used for determining filter parameters and were thus not included in the overall performance metrics (see Section 5). All processing was performed on a Dell Latitude E5550 laptop, equipped with an Intel i5-5300U CPU and 8 gigabytes of RAM.

**Figure 6:**
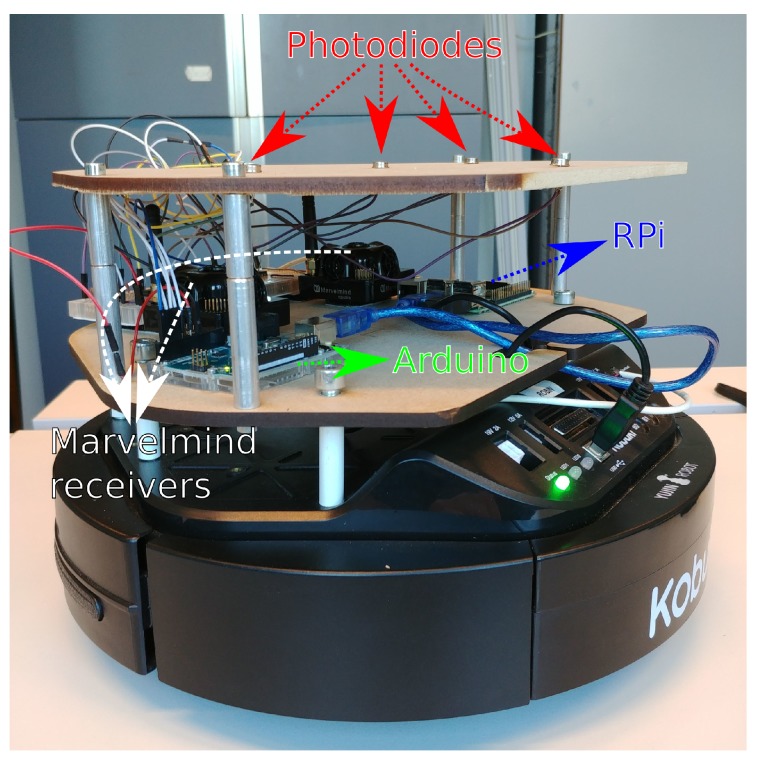
Mobile robot with sensor platform.

A Kobuki robot [32] was selected as the mobile platform (see Figure 6). Several photodiodes were placed on the topmost platform and were connected via a TIA to the sampling pins of an Arduino UNO. This microcontroller relays the light intensity values to a Raspberry Pi (RPi), which is also connected to the robot. The different sensors are synchronized offline. Only the measurements whose timestamps are close enough together are considered. Besides receiving sensor data, the RPi also controls the robot by sending velocity commands. During experiments, a constant forward velocity was applied as a reference to the open-loop speed controller of the robot during each experiment, which enabled it to approximately follow the same trajectory in subsequent experiments. These trajectories were the same as those used in simulations (see Figure 4). The forward speed of the robot was approximately 0.2 m/s.

**Figure 7:**
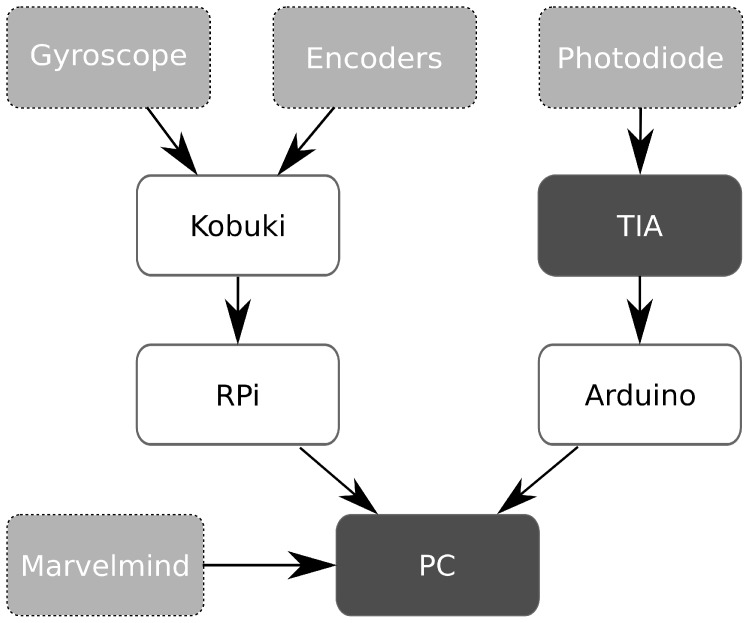
Schematic overview of the hardware used in experiments.

Four LED lights were mounted to the ceiling as transmitters, the model and manufacturer of the LEDs and other components are listed in Table 1. The transmitters were powered by a lab bench power supply. As a ground truth reference (that is, the real location of the robot), the ultrasound positioning system by Marvelmind Robotics was used. We expected the accuracy of our approach to be an order of magnitude lower (that is, a higher error value) than the Marvelmind accuracy of 2 cm [33]. The system was therefore a suitable ground truth reference. The beacons of the Marvelmind system were placed next to the LEDs at a known distance. Through the self-calibration function of the Marvelmind system, we could therefore obtain both beacon and LED coordinates. Finally, a separate computer was also present for visualization, data recording, and sending teleoperation commands via the keyboard. A schematic representation of the system can be seen in Figure 7.

## 4. Simulation Results

In our previous work, we provided baseline simulation results for the two standard trajectories. Complete results can be found in [28], and we only briefly mention the conclusion to provide proper context for the following sections.

An important issue that arises when using unmodified light sources is that a single intensity value does not define a unique location, which may result in data association errors when using a Kalman filter. For this particular lighting layout, these types of errors are largest in the center of the room as the light intensity is initially identical in all directions moving out from the origin. Using multiple receivers significantly reduces the data-association problems observed when using only one receiver. As expected, the overall observed accuracy is also higher when using multiple receivers. Placement of the photodiodes on the robot is important, however. Accuracy improves as the diodes are placed further apart (up to a certain point). Additionally, one should have some receivers which are not aligned with the direction of motion. Therefore, the layout shown in Figure 2 was used for subsequent simulations and experiments using multiple receivers.

### 4.1. Single Receiver Results

#### 4.1.1. Convergence

**Figure 8:**
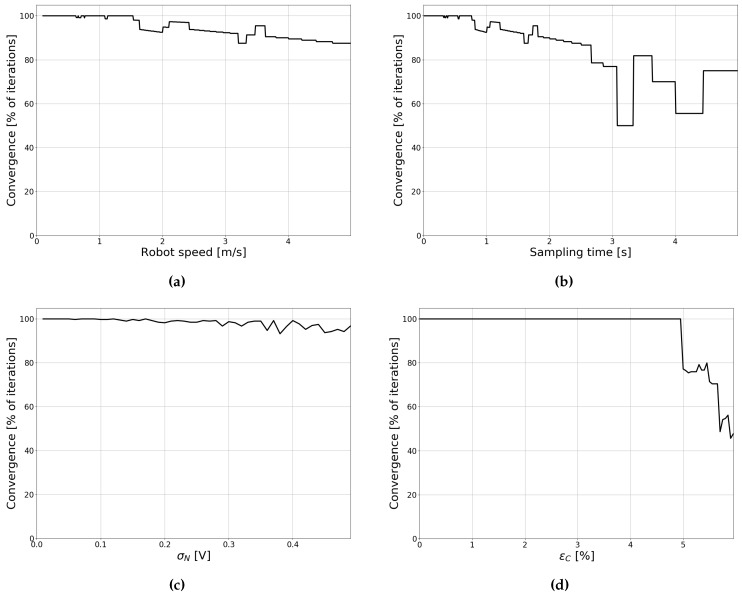
Filter convergence for different parameters. (**a**) Convergence as a function of robot speed, (**b**) Convergence as a function of sampling rate, (**c**) Convergence as a function of measurement noise, and (**d**) Convergence as a function of calibration error.

**Figure 9:**
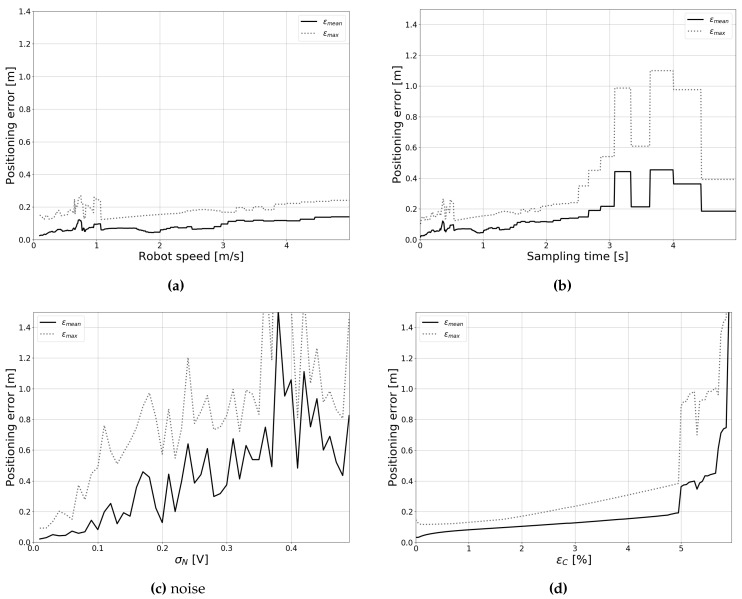
Positioning errors for different parameters. (**a**) Positioning error as a function of robot speed, (**b**) Positioning error as a function of sampling rate, (**c**) Positioning error as a function of measurement noise, and (**d**) Positioning error as a function of calibration error.

The IEKF makes local iterations of the update step, which terminate either when the estimate is converged (that is, the difference between subsequent estimates is lower than a certain threshold), or when a maximum number of iterations is reached. We investigated the influence of certain parameters on filter convergence and accuracy. To this end, we simulated positioning along Path 1 for different parameter values, and recorded the results. Following simulations, we checked how many of the filter updates were deemed converged for each parameter value (that is, the maximum number of iterations was not reached). Figure 8 shows the rate of convergence as a function of different parameters. Additionally, Figure 9 shows the mean and maximum positioning errors during the trajectory for each parameter value. The positioning error was defined as the Euclidean distance between the true position and the estimated position:

(7) $$\varepsilon_{R}=\sqrt{{\left(x_{est}-x_{real}\right)}^{2}+{\left(y_{est}-y_{real}\right)}^{2}}$$

where $\varepsilon_{R}$ is the total positioning error and subscripts est and real are used to denote the estimate and the real coordinates, respectively.

As can be seen in Figure 8a, convergence is initially unaffected by the robot speed. However, when the forward speed becomes higher than approximately 1.5 m/s, the rate of convergence starts the decrease almost linearly. As the speed increases, the displacement of the robot between timesteps increases. Therefore, the filter may take longer to reach a local minimum. Figure 9a shows that increasing the speed also increases the mean and maximum positioning errors, albeit at a relatively slow rate. Increasing the sampling time has similar effects to increasing the robot speed. As the time between samples becomes longer, the displacements between timesteps again becomes larger. As a result, convergence decreases (Figure 8b) and positioning errors increase (Figure 9b). When the sampling rate becomes larger than 3 s, the filter response becomes increasingly unstable. Past a certain point, displacements between estimates become too large, and a local minimum is challenging to obtain.

Figure 8c shows the effect of increasing the measurement noise on filter convergence. Compared to increasing the speed or sampling time, the effect is much less pronounced. On the other hand, when we consider accuracy (Figure 9c), it is clear that increasing the noise has a large effect. These observations are not unexpected, as the rate of convergence simply measures the ability of the filter to find a local optimum. When measurement noise is increased, convergence is still reached rather quickly. Nevertheless, when large amounts of noise are present, it becomes increasingly likely that this optimum is not the correct location, and therefore accuracy decreases.

In Figure 8d and Figure 9d, we study the influence of an imperfect model constant (*C* in Equation (6)). Parameter errors are discussed in more detail in Section 4.4, yet we want to discuss the constant here as this parameter has the largest impact on convergence. Initially, the IEKF does not need a lot of local iterations. Similar to increasing the noise, a local minimum is found rather quickly, yet this is not necessarily the correct location, as can be observed from the increase in positioning errors. However, when the model constant error reaches 5%, performance quickly declines. At this point, the difference between the model and the measurement becomes too large, and the innovation becomes incorrect. Additionally, the covariance matrix R is still relatively small, and this incorrect value is given a large weight, resulting in incorrect positioning updates. Therefore, it is important that the model is calibrated accurately. Section 5.2 discusses the calibration in more detail.

#### 4.1.2. Shadowing of the Receiver

**Figure 10:**
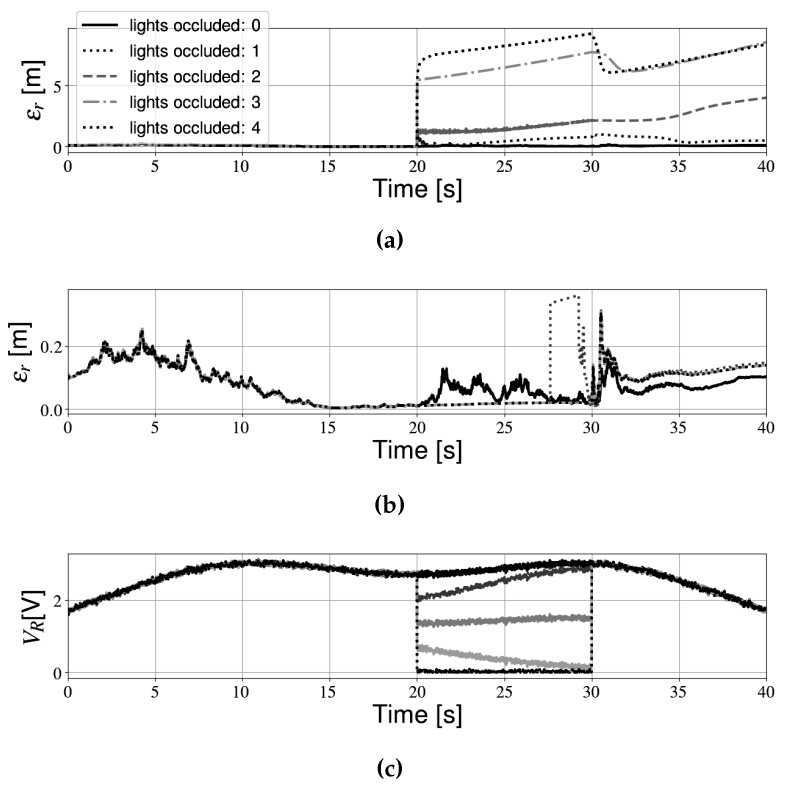
Partial shadowing during Path 1 with an innovation bounds test: (**a**) Total positioning error without innovation bounds test, (**b**) Total positioning error with innovation bounds test, and (**c**) Receiver measurements.

Some effects of data-association errors are mentioned above. In those simulations, this phenomenon resulted in relatively small positioning errors. However, data-association problems can result in much larger positioning errors as well, for example in the case the receiver temporarily detects no light intensity (e.g., when a shadow is cast over the photodiode). The IEKF expects that light intensity measurements are continuously available. When shadows are not accounted for, large differences between the expected values from the model and the measurements from the sensor are observed, which in turn can result in large positioning errors. In the following sections, we investigate the influence of different levels of occlusion (that is, the amount of line-of-sight connections that are broken between transmitters and receivers). In [28], an innovation magnitude bounds test was proposed as a method to compensate occlusions. In the case of a total occlusion (that is, the receiver detects no LOS components), the test provided satisfactory results. However, it is also possible that only one or two line-of-sight components are unavailable, (e.g., when a person walks in front of the robot blocking the view to a light source). These different scenarios each have significantly different effects on the position estimate. Figure 10 shows the total positioning error when one or more light sources are occluded.

Figure 10a shows the total positioning error, and Figure 10c shows the receiver measurements for different levels of occlusion. It is clear that, when more light sources become occluded, the total positioning error increases. As more LOS components are blocked, the measurement model becomes increasingly inaccurate. when only one line-of-sight component is unavailable, the total positioning error increases steadily up to approximately 1 m. When the LOS component becomes available again, accuracy improves, although some residual error remains. In all other cases, however, the position estimate quickly deteriorates. In the case all lights are occluded, the error can reach almost 10 m. Similar results are obtained for the second trajectory, but are not shown here for the sake of simplicity.

#### 4.1.3. Innovation Magnitude Bounds Test

In the case the receiver is unexpectedly completely occluded, the difference between the predicted and the actual measurement is large. As a result, the innovation ν will also have a large value, which is only permitted in the case the covariance of the innovation *S* is also big. It is therefore relatively straightforward to reject unwanted large updates by checking if *v* is bounded by $\pm 2\sqrt{S_{n}}$, which is referred to as an “innovation bounds test” [34]. In the case the innovation is out of bounds, the IEKF reverts back to dead-reckoning until the innovation is again within bounds.

Figure 10b shows the estimation results for the same partial shadowing simulations as before, this time with an innovation bounds test. It is clear that this addition relaxes the LOS requirements. When only one LOS is blocked, the positioning error briefly spikes as the received measurements approach the model. When the same simulations are performed for Path 2, however, the total positioning error reaches a peak of approximately 1.5 m, before dropping back to normal levels. In all other cases, the test is able to reject most incorrect updates during the occlusion. Therefore, we can conclude that the addition of an innovation bounds test makes the filter more robust. One could take a smaller acceptance range in order to improve filter performance. For example, when using a 1-σ limit for Path 2, the total positioning error peaks at 1.1 m instead at 1.5 m with a 2-σ limit. However, by making the acceptance region for the innovation smaller, useful information is also removed, therefore decreasing filter performance in normal conditions. A trade-off can thus be made between robustness in the presence of occlusions, and accuracy in LOS conditions.

### 4.2. Multiple Receiver Results

**Figure 11:**
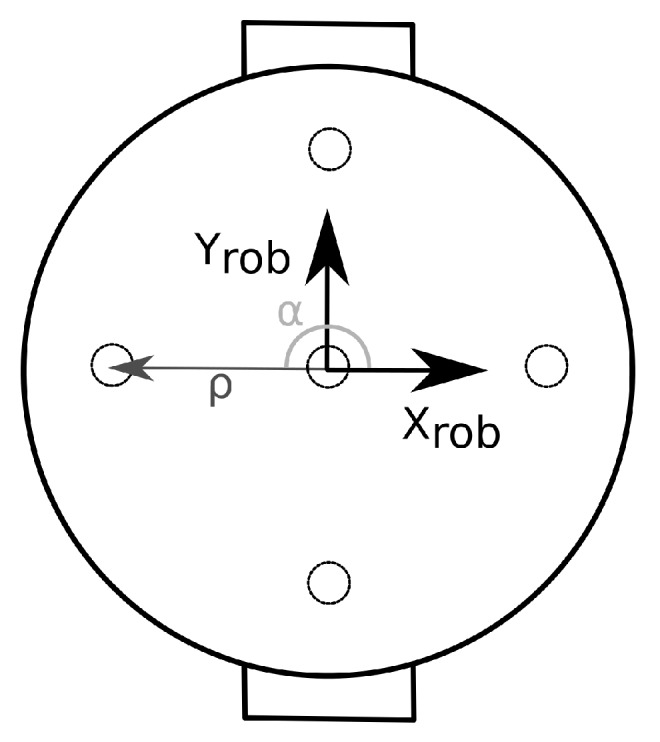
Example configuration with multiple receivers. Dashed circles represent the locations of photodiodes.

The addition of an innovation bounds test makes the filter more robust, but not more accurate. New information is not gained; rather, unexpected measurements are ignored. However, by employing multiple receivers, more information does become available. Including multiple receivers is relatively easy from a hardware point of view, as photodiodes are inexpensive. When multiple receivers are used, both the measurement y and the predicted measurement h(x) become vectors instead of scalar values (as discussed in Section 3.1). In [28], we proposed a multi-receiver configuration whereby one photodiode is placed in the center, and four additional ones are mounted at the edge of the robot at regular intervals (see Figure 11).

#### 4.2.1. Shadowing of Multiple Receivers

Section 4.1.2 discusses the influence of occlusions for a single receiver. Similar simulations can be performed for multiple receivers. For the sake of brevity, Table 3 collects the most important results from simulations with various settings. The table shows for which settings and scenarios the position estimation deteriorates, which is defined as a total positioning error of more than 1 m that does not decrease by the end of the trajectory. Both the default filter and the addition of the innovation bounds test were simulated.

It is clear that, when multiple receivers are used, an innovation bounds test is still required to compensate for occlusions. Similar to the single-receiver simulations, the position estimate now deteriorates when a single light source is occluded during the second trajectory. In this case, the model deviates little from the measurements, and the filter is no longer able to accurately estimate the robot position. This underlines the previous conclusion that this is the most challenging scenario. In all other cases, however, the innovation bounds test successfully rejects faulty measurements.

### 4.3. Random Trajectories

The previous sections focus on filter performance for two standard trajectories, which were selected as they provided both an easy and a more challenging test case. However, positioning also needs to be possible in more general cases. To that end, we generated 100 semi-random trajectories as follows:

1. Obtain the total number of iterations as: $n=\frac{L}{v*T}$, where *L* is the length of the trajectory, *v* is the forward speed of the robot, and *T* is the sampling time.
2. Obtain the initial pose. For all trajectories, the initial position coincides with the origin. Half of the poses were initialized with a heading angle θ of 0 degrees, the other half have an orientation of 180 degrees.
3. Select the number of turns ($n_{turn}$) in the trajectory as a random number between 0 and 10.
4. Calculate the angle that will be covered in each turn (α), which is a random number between −30 and 30 degrees.
5. Determine the angular displacement between estimates (Δθ), such that the angle α is covered in $n/n_{turn}$ iterations.
6. Determine the displacement between estimates as a random number between 0 and $\frac{L}{n}$.

**Figure 12:**
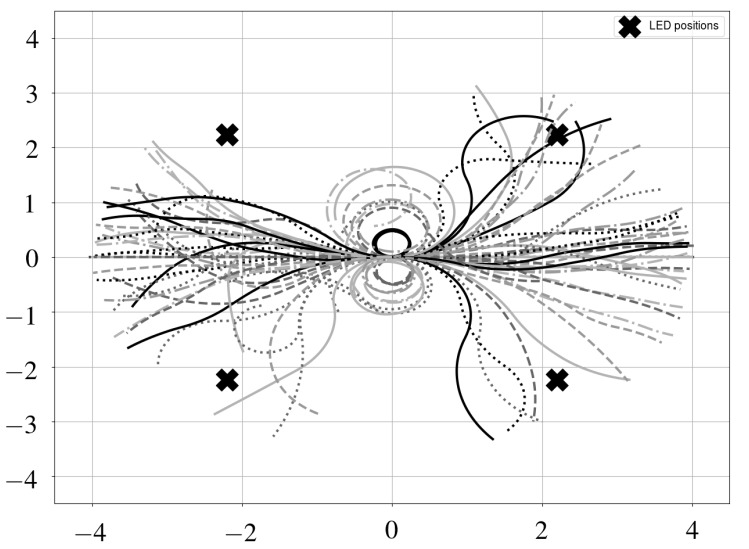
Simulated random trajectories.

**Figure 13:**
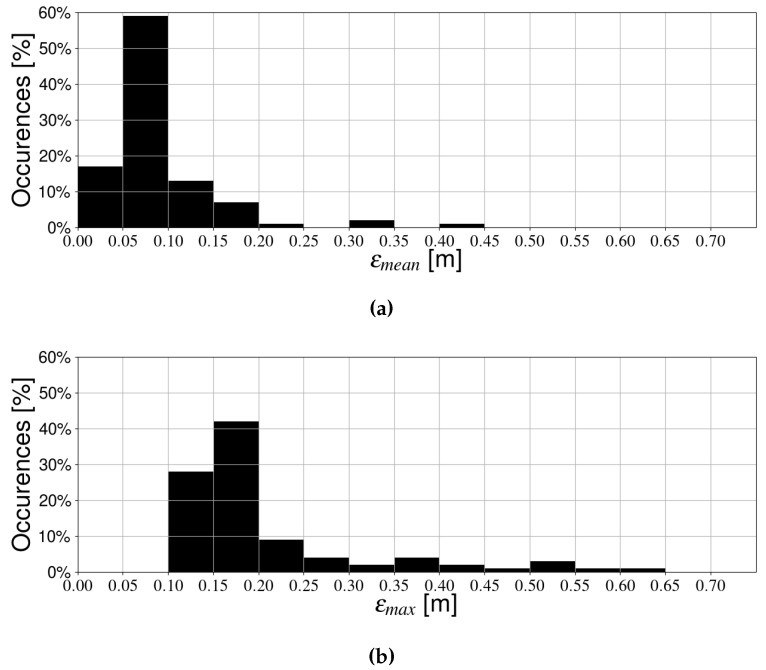
Positioning error during random trajectories when using one receiver. (**a**) Mean positioning error, and (**b**) Maximum positioning error.

**Figure 14:**
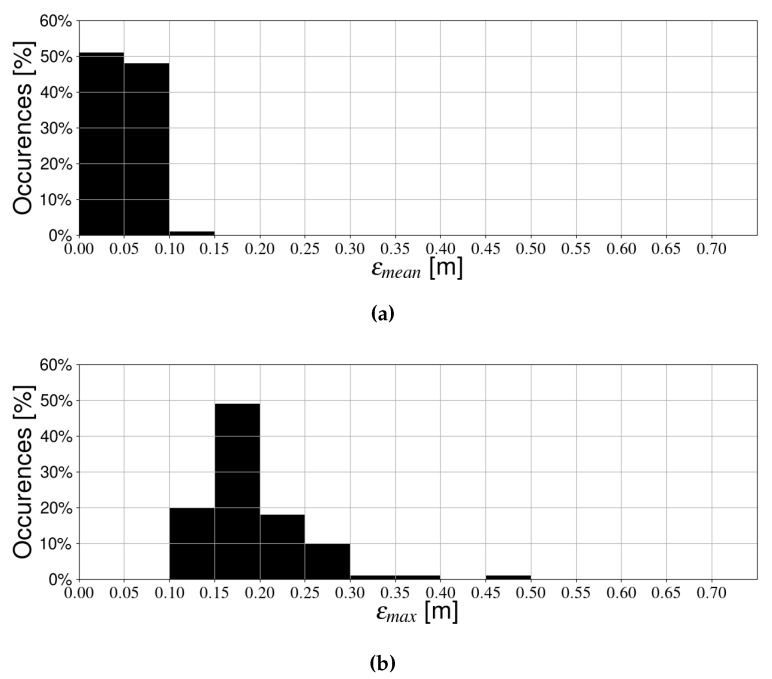
Positioning error during random trajectories when using multiple receivers. (**a**) Mean positioning error, and (**b**) Maximum positioning error.

The resulting trajectories are shown in Figure 12. For every trajectory, we simulated position estimation with one and multiple receivers, and recorded the mean and maximum errors. In Figure 13, results for a single receiver are shown. In the vast majority of cases (around 90%), the mean error is around 15 cm or less. Additionally, most results are clustered relatively close together, although some outliers show significantly higher errors. This effect is magnified in the maximum positioning error, which is even more spread out, indicating that the single receiver case is not very robust. In contrast, Figure 14 shows the mean and maximum positioning errors for all random trajectories when using multiple receivers. Accuracy is much better overall. Moreover, the results are clustered much more closely together, and few extreme outliers can be observed. Therefore, this confirms our previous hypothesis that the multi-receiver layout provides more accurate and robust positioning compared to a single photodiode. Additionally, results are comparable to previous simulations with the standard trajectories, indicating that they are valid test cases.

### 4.4. Parameter Errors

It is important to note that previous simulations assumed that the parameters of the environment (such as the height of the ceiling, orientation of the receiver, etc.) were known exactly. In a real environment, this assumption is not always valid. We therefore investigated the effects of imperfect parameter knowledge. We performed simulations similar to those presented above; however, this time there was a difference between the parameter values accessible to the filter (representing our knowledge of the environment) and their actual values (used to, for example, compute receiver measurements). The difference between the real and the estimated parameter values used in the simulation are collected in Table 4. These values were chosen to represent accuracies that can reasonably be obtained with low cost measurement equipment. A complete sensitivity analysis, whereby the contribution of errors on each individual parameter is investigated, is outside the scope of this paper. This simulation is included not for quantitative accuracy; rather, it is intended to provide an order of magnitude comparison between regular filter behavior and the case of imperfect calibration. Table 5 shows the scenarios for which the filter deteriorates when parameter errors are present, similar to Section 4.2.1. However, contrary to the previous section, the table now contains receiver layouts for which the estimate deteriorates (compared the blocked LOS components in Table 3). Again, both the default filter and the addition of the innovation bounds test are simulated.

Interestingly, deterioration only occurs when a single receiver is used for the second trajectory. The innovations bounds test is unable to compensate for filter divergence in this case. Once again, the second trajectory proves to be a more challenging test case. As expected, multiple receivers provide better and more robust results compared to using only a single receiver. The position estimate never deteriorates when multiple receivers are used. However, it is worth noting that positioning errors are significantly larger than before. The mean error now ranges from 25 to 33 cm, compared to less than 10 cm in previous sections.

## 5. Experimental Results

### 5.1. Overview

Based on the simulations that were performed (see Section 4), a few cases can be defined for experimental evaluation:

- **Dead reckoning**: Only odometry measurements were used for position estimation. These results mainly provide a comparison for the estimates that include light measurements. Overall filter performance depended on the accuracy of dead reckoning. Hence, mainly the improvement over this case was of interest.
- **1 receiver**: Light intensity measurements from a single photodiode were used. This represents the simplest possible case for our proposed approach.
- **5 receivers**: Light intensity measurements from five photodiodes were used for position estimation.

Table 6 provides an overview of the experimental results. Several datasets were collected; three and five experiments were performed for Paths 1 and 2, respectively. One dataset from Path 2 was taken as a calibration dataset to determine the model and filter parameters (see Section 5.2). This dataset was excluded from the validation. The final result in Table 6 was obtained by averaging the results of the individual experiments. Both the mean and the P95 errors are listed. The P95 error is defined as the 95th percentile of the cumulative error distribution. Additionally, Table 6 shows the processing delay for every dataset, which is calculated by dividing the time needed to process all measurements by the number of pose estimates. With this delay, it is possible to calculate the maximum update rate of the algorithm. This rate is limited by the measurement speed of the fastest measuring sensor. In our work, odometry measurements could be obtained at a rate of 50 Hz, while the light intensity could be sampled at approximately 130 Hz. The maximum update rate possible could then be obtained by:

(8) $$f_{update}=\frac{1}{\tau_{sensor}+\tau_{processing}}$$

where $f_{update}$ is the rate of position estimation, $\tau_{sensor}$ is the delay of the fastest sensor in the loop, and $\tau_{processing}$ is the processing delay. A can be seen in Table 6, in dead reckoning, data processing adds no appreciable delay. The maximum update rate is lower compared to the IEKF, however, as the light measurements can be obtained at higher rates. The following sections discuss these results in more detail.

### 5.2. Verification of the Measurement Model

**Figure 15:**
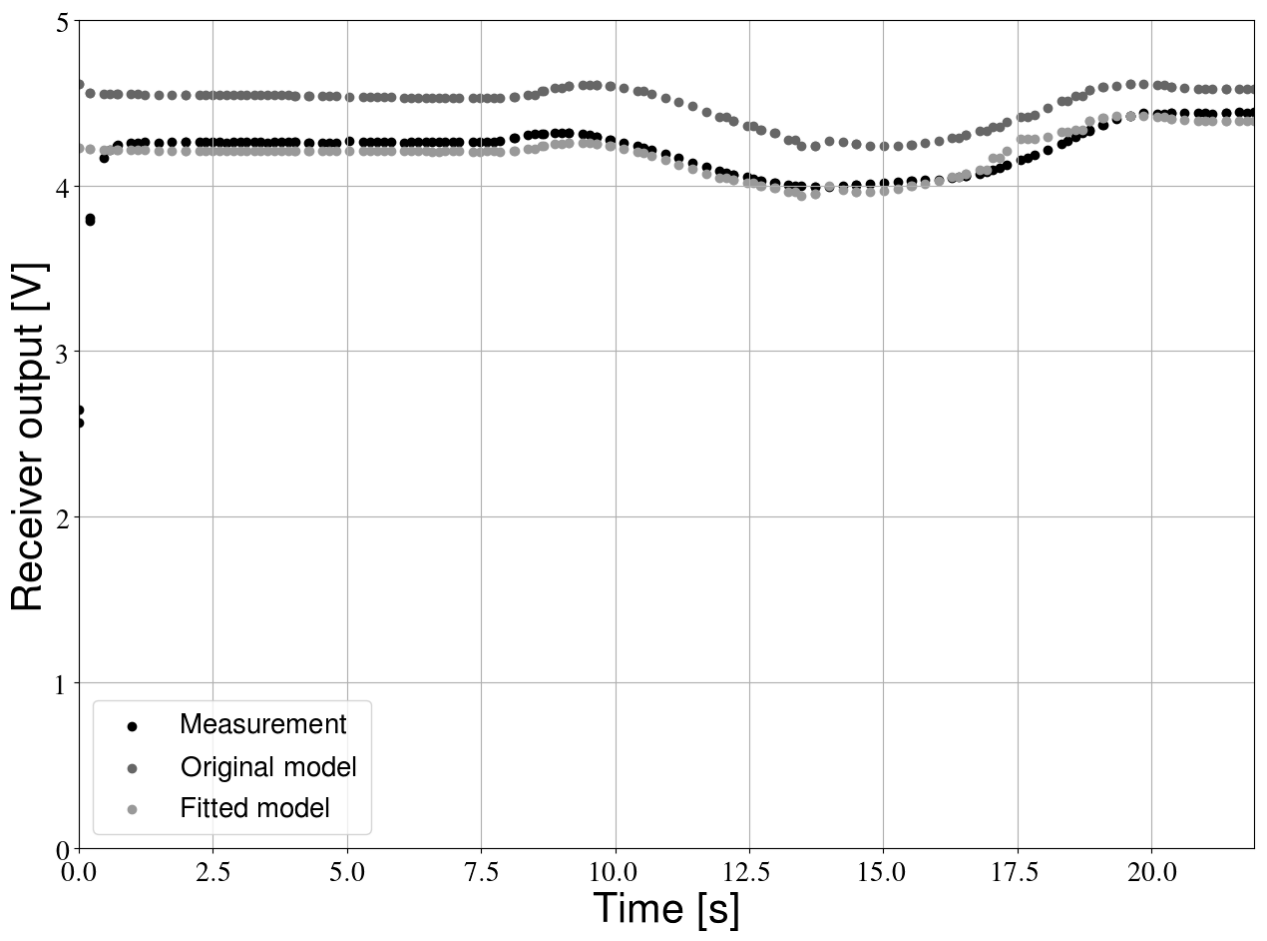
Light intensity measurements with calibrated model.

Before the proposed positioning approach could be benchmarked experimentally, it was important to first verify if the measurement model is accurate. To this end, the robot was placed inside the test environment. The mobile platform was operated remotely and light intensity data as well as the ground truth position of the robot were recorded. In post-processing, the light intensity was predicted by the measurement model at the ground truth positions (see Figure 15). The measured intensities were first passed through a Butterworth low-pass filter to reduce noise.

A relatively large difference between the model and the measurements can be observed. This difference is mainly due to the model constant *C* (see Equation (6)), which is a parameter that collects all the non-position dependent values. This constant is relatively difficult to predict beforehand (e.g., the resistor values have some offset, the emitted optical power is not perfectly well known, etc.). While knowledge of the components of *C* is not needed for other parts of the positioning algorithm, we do need to know their combined value for localization. As shown in Section 4.1.1 and Section 4.4, it is important to know the model constant relatively accurately. A one-time calibration was therefore necessary. During this procedure, the values of *C* for every receiver were determined by fitting the measurements to the model with a non-linear least-squares fit. In this work, we only calibrated the model constants. Attempts at simultaneously fitting the Lambertian emission order *m* or the transmitter height resulted in overfitting and consequently in large positioning errors. The scaled model shown in Figure 15 provides a much better fit. The parameters of the scaled model were used to obtain the experimental results in the following sections.

### 5.3. Initial Position Estimate

**Figure 16:**
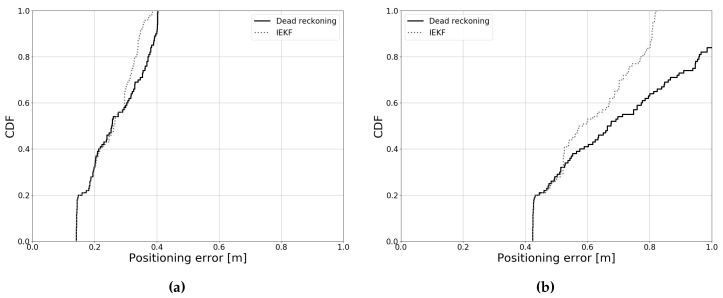
Error distribution for different initial estimates. (**a**) Initial error of 10 cm per coordinate, (**b**) Initial error of 30 cm per coordinate.

As the Kalman filter uses data from both the previous and the current timestep, the results are heavily influenced by the initial estimate. This is illustrated in Figure 16, which shows the cumulative error distribution for a certain experiment. The *x*-axis shows the positioning error and the *y*-axis shows the normalized occurrences of that value. In Figure 16a, the initial error on the *x*- and *y*-coordinates is 10 cm, relative to the ground truth. It is clear that, in this case, the IEKF provides only a relatively small improvement in the higher intervals of the probability distribution. In Figure 16b, the initial error is 30 cm on both coordinate axes. As expected, the overall accuracy decreases for both dead-reckoning and the Kalman filter. However, one can also observe that the relative improvement of the IEKF is much greater compared to Figure 16a. This observation is not specific to our use case; rather, it is a general property of the Kalman filter. In the following sections, the error on the initial estimate will be 20 cm per coordinate axis, unless otherwise specified.

### 5.4. Single Receiver Results

During experiments, intensity data of five photodiodes were recorded. In this experiment, the data from only one of these receivers were passed on to the filter. The photodiode was in this case located in the center of the robot, as in Section 4.1. This enabled testing the algorithm in its most basic implementation, similar to the simulation results.

**Figure 17:**
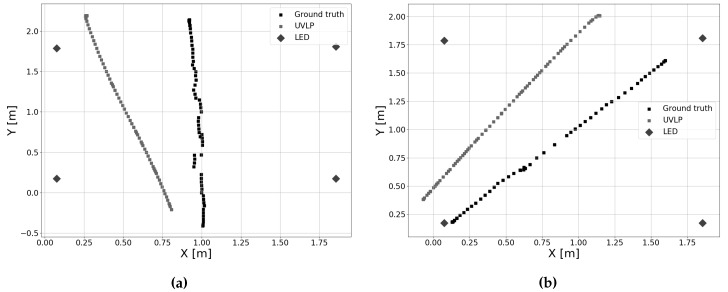
Position estimates with ground truth when one receiver is used. (**a**) Position estimate for Path 1, (**b**) Position estimate for Path 2.

**Figure 18:**
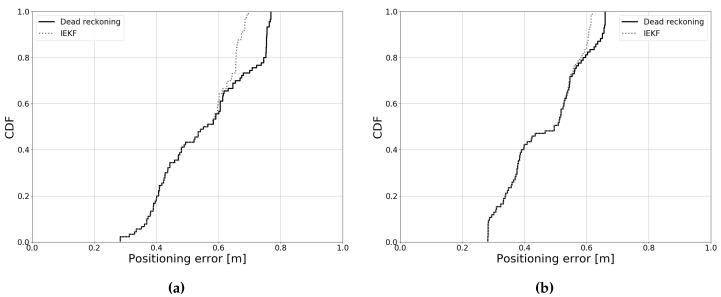
Cumulative error distributions when one receiver is used. (**a**) Error distribution for Path 1, (**b**) Error distribution for Path 2.

Figure 17 shows example outputs of experiments where the robot was driven along Paths 1 and 2. Only the estimates with timestamps approximately matching the ground truth measurements are used for evaluation (a tolerance of 10 ms was allowed). In Table 6, it is clear that, in the case of one receiver, little improvement is present over dead reckoning. The total positioning error increases over time, as would be the case when only odometry measurements are used. Indeed, Figure 18 shows that only in the very highest intervals of the cumulative error distribution, a slight improvement can be observed. This is in contrast with our simulations, and can likely be attributed to the greater error on the initial angle, which cannot be corrected by using one receiver in the center of the robot. Additionally, reflections from the white walls and reflective metal surfaces in the setup (see Figure 5), as well as imperfect calibration likely also contribute to the increased error. Overall, it is challenging to remove the bias (that is the initial positioning error) by using data from one receiver.

Table 6 also lists the update rate of the IEKF and dead reckoning. When only odometry measurements are used, computational requirements are extremely low, and data can be processed almost instantly. The addition of the update step does significantly increase processing time. However, light intensity can be captured at a much higher rate (130 Hz compared to 50 Hz for odometry). Additionally, the processing delay is significantly lower than the measurement delay. Therefore, the update rate of the IEKF is actually higher than dead reckoning.

### 5.5. Multiple Receiver Results

**Figure 19:**
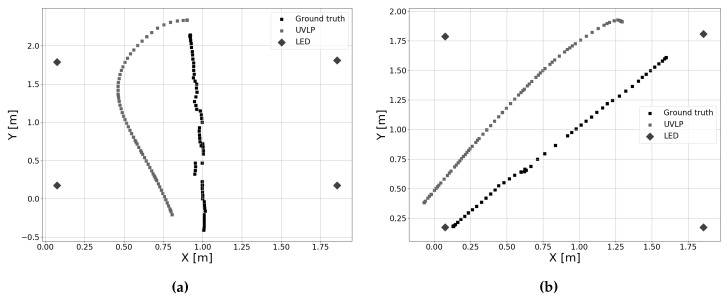
Position estimates with ground truth when five receivers are used. (**a**) Position estimate for Path 1, (**b**) Position estimate for Path 2.

In the experiments in Section 5.4, measurements from only a single receiver were used. In this experiment, measurements from all five photodiodes were used in position estimation. As in Section 5.4, Figure 19 shows the position estimates for the defined trajectories, along with the ground truth. Contrary to the case of a single receiver, the IEKF is now able to make more and better corrections of the estimate. This is especially noticeable in Path 1 (Figure 19a). The estimate initially deviates, yet after some time starts to converge towards the true position. This effect is also present in Figure 19b, yet to a lesser extent. In both cases, some error does remain on the pose estimate, as the approach cannot estimate the exact location.

**Figure 20:**
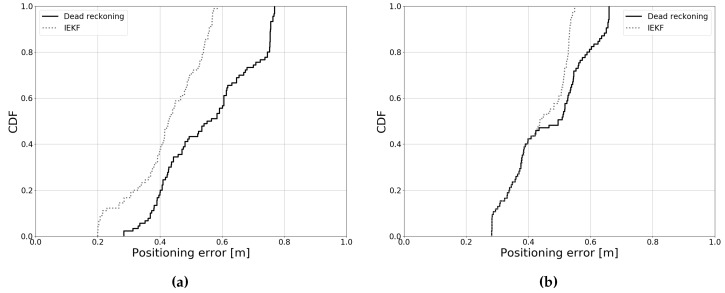
Cumulative error distributions when five receivers are used. (**a**) Error distribution for Path 1, (**b**) Error distribution for Path 2.

Figure 20 shows the cumulative error distribution for both trajectories compared to dead-reckoning. For Path 1, a relatively large improvement can be observed. For Path 2, this improvement is more modest, yet significantly higher than the case of one receiver (Figure 18b). As can be seen in Table 6, this improved accuracy comes at the cost of a lower update rate. The increased size of the Jacobian matrix and additional measurement predictions both increase the computational complexity. The update rate remains high, however.

## 6. Discussion

The accuracy we obtained is significantly lower than some RSS based VLP systems, which can be in the range of a few centimeters [11]. However, we do not require any modulation hardware for the transmitters. Additionally, multiplexing techniques that are needed for photodiode based receivers [19] are also not needed. Using only existing light sources thus simplifies hardware requirements, at the cost of accuracy.

Previous works have already investigated the use of probabilistic filters for positioning with unmodified light sources. Xu, Zheng and Hranilovic [27] obtained an average accuracy similar to our work. Depending on the layout of the building, the mean accuracy is between 0.38 m and 0.74 m. However, their algorithm works on a commercial smartphone, which is arguably a more challenging use case than a mobile robot. Encoders generally provide more accurate motion data compared to accelerometers. The Lightitude system developed by Hu et al. [26] achieves a considerably lower accuracy of approximately 2 m. However, their test setup is larger than our lab scale environment. Finally, Jimenez, Zampella and Seco developed their Light-Matching system and tested it with both simulations [35] and experiments [25]. Their approach is able to achieve an accuracy of around 1 m on average. Similar to Xu et al. [27] and Hu et al. [26], a smartphone is used to collect light intensity measurements. However, the IMU is mounted on the foot, which enhances performance yet is less practical for the user. Thus, while the accuracy of our work is comparable or better than similar works in the literature, one should keep in mind that our environment is smaller. Therefore, mainly the gain over dead reckoning is of importance. How large the impact is depends on the initial estimate, the followed trajectory, and the number of receivers. From the results, it is clear that an improvement is always present, however. The best results are obtained when multiple photodiodes are used at different locations on the moving platform. This favors both robustness and accuracy of the position estimation.

The main advantage of an IEKF is the reduced computational load. Particle filters typically need to update the probability of many possible estimates; therefore, the prediction and measurement models outlined in Section 3.1 need to be calculated many times. By using a Kalman filter, the computations only need to be performed a few times. The position estimate can therefore be updated more frequently, which is interesting for highly dynamic applications. Alternatively, one could use the newly available resources elsewhere, for example for autonomous navigation of mobile robots. Finally, one could also take advantage of the reduced complexity by simply prolonging the battery life of the device.

Our approach requires an initial position estimate; therefore, it would be useful to combine this work with one of the particle filters proposed in the literature. One of the characteristics of the filter is the ability to localize a user without knowledge of the initial conditions. Initially, a uniform distribution of particles over the entire floor could be used for the particle filter. When more data are gathered, the particles start to converge to one or more estimates, as certain hypotheses become impossible. Once the particles are converged to a single cluster, a switch could be made to the Kalman filter for a decrease in computational power. One could then monitor whether the observations of the Kalman filter match the model (e.g., through an innovation bounds test) to detect a divergence in the position estimate. A switch back to the particle filter could be made if necessary. This would result in a system that is both robust and efficient. A similar suggestion was made by Gutmann et al. for robot localization based on laser scan matching [36]. The general principle could be applied to UVLP as well. Alternatively, UVLP could be combined with standard VLP. A select number of light sources can be outfitted with modulation hardware. Once a modulated source is observed, it provides a global location fix with which we can initialize the Kalman filter. Increasing the amount of VLP sources increases accuracy and robustness, yet also increases cost. Depending on the application, a trade-off can therefore be made. The accuracy of UVLP may not be sufficient for all types of robot positioning. Instead, another application for UVLP would be asset tracking, for example in warehouses and hospitals. A survey on the benefits of indoor location identified an accuracy of 1 m to be sufficient for a large number of use cases [37].

## 7. Conclusions

In this work, we outline a computationally efficient method for positioning based on unmodulated light. We demonstrated its capabilities through both simulations and experiments. The accuracy is dependent on a number of factors, yet is below 0.5 m on average in most cases. In the best case, the average accuracy can be around 0.4 m during experiments. Due to the low computational complexity, the algorithm is able to estimate the pose of the robot at rates between 84 and 112 Hz. Additionally, occlusions can mostly be compensated. Only when the expected and true measurements are close together do problems arise. The main drawback is the need for an initial position estimate. In future work, we could combine our approach with a particle filter from literature, to relax this requirement. Alternatively, the system could be integrated in an indoor space with sparsely distributed standard VLP sources. The modulated lights can provide a global location fix for Kalman filter initialization. A trade-off can be made between increasing the accuracy and reducing the complexity and cost of the system. Ultimately, light intensity by itself is a relatively sparse source of information, and should be combined with other sensor data (e.g., IMU, encoder, WIFI, modulated VLP, etc.) to obtain the best results. In future work, the possibilities of UVLP as a low-cost tracking technology in such use cases should be explored as well.

## Figures and Tables

**Table 1:** Overview of the main hardware components.

Component	Manufacturer	Model Name
LED	Bridgelux	BXRC-50E4000-F-24
Photodiode	Osram	BPX 61
Robot platform	Yujin Robot	Kobuki
Microcontroller	CC Logistics LLC	Arduino UNO
Embedded board	Raspberry Pi 3	Raspberry Pi Foundation
Ground truth location reference	Starter set (433 MHz)	Marvelmind Robotics

**Table 2:** Simulation parameters.

Symbol	Description	Value	Unit
**Receiver parameters**			
*A*	Area of receiver	7.02	mm^2^
*G*	TIA Gain	500	kOhm
$R_{P}$	Peak responsivity of photodiode	0.62	A/W
$g_{m}$	FET transinductance	30	mS
Γ	FET noise factor	1.5	/
η	Capacitance per unit area	1.026 × 10^−11^	F/mm^2^
$I_{2}$	Noise bandwidth parameter	0.562	/
$I_{3}$	Noise bandwidth parameter	0.0868	/
$I_{bg}$	Background current	190	μA
$B_{m}$	Equivalent noise bandwidth	100	MHz
**Transmitter parameters**			
$\phi_{v}$	Luminous flux	4275	lm
*m*	Order of Lambertian emission	1	/
$\phi_{1/2}$	Half power angle	60	${}^{\circ }$
CCT	Correlated color temperature	5000	K
	Position LED${}_{1}$ [x,y]	[−2.20, −2.235]	m
	Position LED${}_{2}$ [x,y]	[2.20, −2.235]	m
	Position LED${}_{3}$ [x,y]	[2.20, 2.235]	m
	Position LED${}_{4}$ [x,y]	[−2.20, 2.235]	m
**Simulation parameters**			
Δt	Duration of time step	0.1	s
$d_{z}$	Height difference of receiver and transmitter	2.8	m
*T*	Ambient temperature	293	K
**Filter parameters**			
$n_{IEKF}$	Maximum number of update iterations	10	/
$d_{min}$	Minimum distance between subsequent IEKF iterations	0.05	m
$\sigma_{x,0}^{2}$	Initial variance on *x*-coordinate	0.04	m^2^
$\sigma_{y,0}^{2}$	Initial variance on *y*-coordinate	0.04	m^2^
$\sigma_{\theta ,0}^{2}$	Initial variance on heading angle	0.05	radians^2^
$\sigma_{\Delta s}^{2}$	Variance on odometry distance measurements	3.60 × 10^−9^	m^2^
$\sigma_{\Delta \theta }^{2}$	Variance on odometry angle measurements	2.50 × 10^7^	radians^2^

**Table 3:** Position estimate deterioration for levels of shadowing with multiple receivers. The numbers in the table indicate the number of LOS components that need to be unavailable for the position estimate to deteriorate.

Trajectory	Default	Innovation Bounds
Path 1	2,3,4	/
Path 2	2,3,4	1

**Table 4:** Simulated parameter errors.

Parameter	Error	Unit
Ceiling height	0.01	m
Receiver angle	1	${}^{\circ }$
Model constant (*C* in Equation (5))	1.5	%

**Table 5:** Position estimate deterioration with parameter errors. Table entries correspond to the receiver configuration that leads filter divergence.

Trajectory	Default	Innovation Bounds
Path 1	/	/
Path 2	Single	Single

**Table 6:** Overview of experimental results

Dataset	Mean Error [m]	P95 Error [m]	Processing Delay [ms]	Maximum Update Rate [Hz]
DR, Path 1	0.438	0.569	0.2	50
One receiver, Path 1	0.406	0.502	1.2	112
Five receivers, Path 1	0.336	0.464	4.3	84
DR, Path 2	0.321	0.408	0.2	50
One receiver, Path 2	0.316	0.390	1.2	112
Five receivers, Path 2	0.298	0.363	4.2	84

## References

[1] Mautz R. (2012). Indoor Positioning Technologies. Ph.D. Thesis.

[2] Bejuri W.M., Wan Y., Mohamad M., Sapri M. (2011). Ubiquitous Positioning: A Taxonomy for Location Determination on Mobile Navigation System. arXiv.

[3] Gu Y., Lo A., Niemegeers I. (2009). A Survey of Indoor Positioning Systems for Wireless Personal Networks. IEEE Commun. Surv. Tutor..

[4] Dabove P., Di Pietra V., Piras M., Jabbar A.A., Kazim S.A. Indoor Positioning Using Ultra-wide Band (UWB) Technologies: Positioning Accuracies and Sensors’ Performances. Proceedings of the 2018 IEEE/ION Position, Location and Navigation Symposium.

[5] Statler S. (2016). Beacon Technologies.

[6] Makki A., Siddig A., Saad M., Bleakley C. (2015). Survey of WiFi Positioning Using Time-based Techniques. Comput. Netw..

[7] Zhuang Y., Hua L., Qi L., Yang J., Cao P., Cao Y., Wu Y., Thompson J., Haas H. (2018). A Survey of Positioning Systems Using Visible LED Lights. IEEE Commu. Surv. Tutor..

[8] (2017). Global LED Lighting Market: Forecast by Applications, Regions and Companies. Technical Report, Renub Research. https://www.researchandmarkets.com/reports/4437008/global-led-lighting-market-forecast-by.

[9] Haas H., Yin L., Wang Y., Chen C. (2015). What is LiFi?. J. Lightware Technol..

[10] Ghassemlooy Z., Popoola W., Rajbhandari S. (2012). Optical Wireless Communications: System and Channel Modelling with Matlab^®^.

[11] Do T.H., Yoo M. (2016). An in-Depth Survey of Visible Light Communication Based Positioning Systems. Sensors.

[12] Yang H., Du P., Zhong W., Chen C., Alphones A., Zhang S. (2019). Reinforcement Learning-Based Intelligent Resource Allocation for Integrated VLCP Systems. IEEE Wirel. Commun. Lett..

[13] Du P., Zhang S., Chen C., Alphones A., Zhong W.D. (2018). Demonstration of a Low-Complexity Indoor Visible Light Positioning System Using an Enhanced TDOA Scheme. IEEE Photonics J..

[14] Claudio F., Tièche F., Hügli H. Self-Positioning Robot Navigation Using Ceiling Image Sequences. Proceedings of the Asian Conference on Computer Vision.

[15] Panzieri S., Pascucci F., Setola R., Ulivi G. (2001). A Low Cost Vision Based Localization System for Mobile Robots. Target.

[16] Wang H., Ishimatsu T., Mian J.T. (1997). Self-Localization for an Electric Wheelchair. JSME Int. J. Ser. C.

[17] Launay F., Ohya A., Yuta S. A Corridors Lights based Navigation System including Path Definition using a Topologically Corrected Map for Indoor Mobile Robots. Proceedings of the 2002 IEEE International Conference on Robotics and Automation.

[18] Chen X., Jia Y. (2014). Indoor Localization for Mobile Robots Using Lampshade Corners as Landmarks: Visual System Calibration, Feature Extraction and Experiments. Int. J. Control Autom. Syst..

[19] Lausnay S.D., Strycker L.D., Goemaere J.P., Nauwelaers B., Stevens N. A survey on multiple access Visible Light Positioning. Proceedings of the 2016 IEEE International Conference on Emerging Technologies and Innovative Business Practices for the Transformation of Societies (EmergiTech).

[20] Ravi N., Iftode L. (2007). FiatLux: Fingerprinting Rooms Using Light Intensity.

[21] Golding A., Lesh N. Indoor Navigation Using a Diverse Set of Cheap, Wearable Sensors. Proceedings of the Third International Symposium on Wearable Computers.

[22] Azizyan M., Constandache I., Roy Choudhury R. SurroundSense: Mobile Phone Localization via Ambience Fingerprinting. Proceedings of the 15th annual international conference on Mobile computing and networking.

[23] Zhang C., Zhang X. LiTell: robust indoor localization using unmodified light fixtures. Proceedings of the 22nd Annual International Conference on Mobile Computing and Networking.

[24] Wang Q., Wang X., Ye L., Men A., Zhao F., Luo H., Huang Y. A Multimode Fusion Visible Light Localization Algorithm Using Ambient Lights. Proceedings of the 5th IEEE Conference on Ubiquitous Positioning, Indoor Navigation and Location-Based Services.

[25] Jiménez A., Zampella F., Seco F. (2014). Improving Inertial Pedestrian Dead-Reckoning by Detecting Unmodified Switched-on Lamps in Buildings. Sensors.

[26] Hu Y., Xiong Y., Huang W., Li X.Y., Zhang Y., Mao X., Yang P., Wang C. Lightitude: Indoor Positioning Using Ubiquitous Visible Lights and COTS Devices. Proceedings of the International Conference on Distributed Computing Systems.

[27] Xu Q., Zheng R., Hranilovic S. IDyLL: Indoor Localization using Inertial and Light Sensors on Smartphones. Proceedings of the 2015 ACM International Joint Conference on Pervasive and Ubiquitous Computing.

[28] Amsters R., Demeester E., Stevens N., Slaets P. Unmodulated Visible Light Positioning using the Iterated Extended Kalman Filter. Proceedings of the 2018 International Conference on Indoor Positioning and Indoor Navigation (IPIN).

[29] Havlík J., Straka O. Performance evaluation of iterated extended Kalman filter with variable step-length. Proceedings of the 12th European Workshop on Advanced Control and Diagnosis.

[30] Thrun S., Burgard W., Fox D. (2005). Probabilistic Robotics.

[31] Kahn J.M., Barry J.R. (1997). Wireless infrared communications. Proc. IEEE.

[32] Yujin Robot KOBUKI|Kobuki|Waiterbot|Shooter. http://kobuki.yujinrobot.com/.

[33] Amsters R., Demeester E., Stevens N., Lauwers Q., Slaets P. Evaluation of Low-Cost/High-Accuracy Indoor Positioning Systems. Proceedings of the 2019 International Conference on Advances in Sensors, Actuators, Metering and Sensing (ALLSENSORS).

[34] Bar-Shalom Y., Li X.R., Kirubarajan T. (2004). Estimation with Applications to Tracking and Navigation: Theory Algorithms and Software.

[35] Jiménez A.R., Zampella F., Seco F. Light-matching: A new signal of opportunity for pedestrian indoor navigation. Proceedings of the 2013 International Conference on Indoor Positioning and Indoor Navigation.

[36] Gutmann J.S., Burgard W., Fox D., Konolige K. An experimental comparison of localization methods. Proceedings of the 1998 IEEE/RSJ International Conference on Intelligent Robots and Systems Innovations in Theory, Practice and Applications (Cat. No. 98CH36190).

[37] Conti G., Malabocchia F., Li K.J., Percivall G., Burroughs K., Strickland S. (2016). Benefits of Indoor Location, Use Case Survey of Lessons Learned and Expectations. Technical Report. https://portal.opengeospatial.org/files/?artifact_id=68604&usg=AOvVaw16eOF9lRQsNqp55F5Ia-M-.

